# Design of Biopolymer-Based Interstitial Therapies for the Treatment of Glioblastoma

**DOI:** 10.3390/ijms222313160

**Published:** 2021-12-06

**Authors:** Erik S. Pena, Elizabeth G. Graham-Gurysh, Eric M. Bachelder, Kristy M. Ainslie

**Affiliations:** 1 Joint Department of Biomedical Engineering, University of North Carolina at Chapel Hill, Chapel Hill, NC 27599, USA; erikpena@email.unc.edu; 2 Joint Department of Biomedical Engineering, North Carolina State University, Chapel Hill, NC 27599, USA; 3 Division of Pharmacoengineering and Molecular Pharmaceutics, Eshelman School of Pharmacy, University of North Carolina at Chapel Hill, Chapel Hill, NC 27599, USA; elgraham@email.unc.edu (E.G.G.-G.); ebacheld@email.unc.edu (E.M.B.); 4 Department of Microbiology and Immunology, University of North Carolina at Chapel Hill, Chapel Hill, NC 27599, USA

**Keywords:** drug delivery, chemotherapy, release rate, hydrogel, electrospun fibers, compression molding, wafer, local delivery, glioma

## Abstract

Glioblastoma multiforme (GBM) is the most common form of primary brain cancer and has the highest morbidity rate and current treatments result in a bleak 5-year survival rate of 5.6%. Interstitial therapy is one option to increase survival. Drug delivery by interstitial therapy most commonly makes use of a polymer implant encapsulating a drug which releases as the polymer degrades. Interstitial therapy has been extensively studied as a treatment option for GBM as it provides several advantages over systemic administration of chemotherapeutics. Primarily, it can be applied behind the blood–brain barrier, increasing the number of possible chemotherapeutic candidates that can be used and reducing systemic levels of the therapy while concentrating it near the cancer source. With interstitial therapy, multiple drugs can be released locally into the brain at the site of resection as the polymer of the implant degrades, and the release profile of these drugs can be tailored to optimize combination therapy or maintain synergistic ratios. This can bypass the blood–brain barrier, alleviate systemic toxicity, and resolve drug resistance in the tumor. However, tailoring drug release requires appropriate consideration of the complex relationship between the drug, polymer, and formulation method. Drug physicochemical properties can result in intermolecular bonding with the polymeric matrix and affect drug distribution in the implant depending on the formulation method used. This review is focused on current works that have applied interstitial therapy towards GBM, discusses polymer and formulation methods, and provides design considerations for future implantable biodegradable materials.

## 1. Introduction

Glioblastoma multiforme (GBM) encompasses 47.7% of all malignant primary brain and central nervous system (CNS) tumors, accounting for 9.23 cases per 100,000 individuals [1]. The current standard of care therapy is resection, radiotherapy, and chemotherapy with temozolomide (TMZ, brand name: Temodar^®^) [2,3]. However, despite this multi-pronged approach, GBM has an exceedingly poor 5-year survival rate of 5.6% [1,4]. GBM’s vast intratumor heterogeneity [4,5,6,7] and high invasive nature leads to 90% of tumors re-emerging within two centimeters from the original site [8]. GBM’s invasiveness makes surgical resection of the local tumor difficult and gives rise to its high mortality rate [8]. Another complication in treating GBM is the lack of viable chemotherapeutic drug options, due to many having limited or poor penetration capabilities through the blood–brain barrier (BBB) [9,10]. The BBB is a protective barrier composed of numerous cell types that line the blood vessels in the brain and acts as a very strict filter between the CNS and the rest of the body [9,10]. While TMZ can cross the BBB (approximately 1.18 ± 0.3% of drug crosses into mouse brain tissue with intravenous injection) [11], its lipophilic properties, low molecular weight, and short half-life require high doses which can cause toxic side effects [2,12,13,14,15]. Drug resistance to TMZ occurs in approximately 50% of patients wherein the tumors increase expression of the DNA repair protein O6-methylgaunine-DNA methyltransferase (MGMT), limiting the effects of the drug [16,17]. Current therapies have only minimally improved the prognosis for GBM patients and as such there is a pressing need for new therapies to overcome this complex tumor.

Interstitial drug delivery is one alternative where a drug is administered locally to the brain at a sustained or controlled rate, thus bypassing the BBB and minimizing systemic side effects. This approach can have the advantage of reducing treatment intervals and increasing drug therapeutic index. With sustained release of a drug locally, therapeutic concentrations can be kept constant directly at the tumor site rather than requiring multiple systemic administrations of drug to reach the same therapeutic levels in the brain tissue. There are, of course, other routes of therapy that utilize polymers to resolve the issues that systemic drug administrations are faced with. One such route is to functionalize the polymer with tumor-targeting capabilities and/or a moiety that can assist with crossing the BBB to deliver a therapeutic [18].

**Figure 1:**
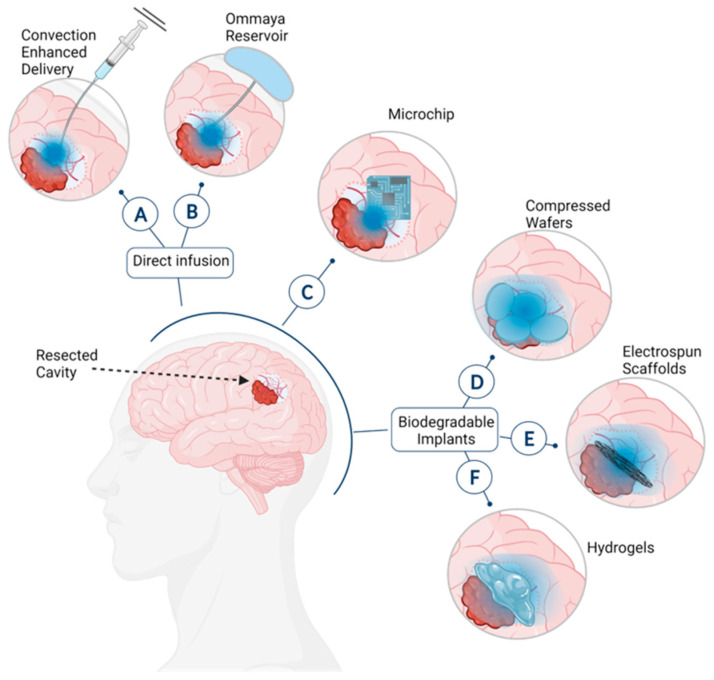
Forms of interstitial drug administration for GBM via (**A**) convection-enhanced delivery, (**B**) Ommaya reservoir, (**C**) microchip, (**D**) drug-loaded compressed wafers, (**E**) electrospun scaffolds, and (**F**) hydrogels.

The simplest approach to interstitial therapy is direct infusion of the drug into the brain. This can be achieved through several methods, such as convection-enhanced delivery, where a drug is delivered via a catheter and driven by a pressure gradient to increase drug distribution throughout the brain (Figure 1A) [19]. The drug can also be loaded into a polymeric particle to be injected into the resection cavity [20]. A similar method is use of an Ommaya reservoir where the drug is stored in a reservoir under the scalp and delivered by a concentration gradient (Figure 1B) [21]. Unfortunately, with these methods there are complications such as infection, catheter blockage, and incorrect placement [22,23,24]. Microchip delivery systems can also be surgically implanted to release a drug upon electrochemical dissolution of an anode membrane (Figure 1C) [25]. Additionally, as microchips are made of silicon, which does not degrade readily, it would require a secondary invasive procedure to remove the device. Another strategy for interstitial therapy is implantation of a drug-loaded degradable biomaterial that is typically administered during the initial resection surgery (Figure 1D–F) [26]. This has the benefit of not requiring a second surgery to remove the drug delivery system. 

Interstitial delivery of drug-loaded degradable polymers can ensure drug concentrations are kept at a safe therapeutic level in appropriate areas of the brain. With these systems, release rate has been shown to have significant impact on therapeutic efficacy [27,28,29,30,31]. However, controlling drug release rate can be complicated as there is a complex interdependent interaction between the drug, polymer, and the formulation method. This affects the profile of drug release, and subsequently drug distribution within the brain which in turn affects therapeutic efficacy. Herein, we review current polymer and formulation methods that have used towards interstitial therapy while discussing the development and limitations of Gliadel^®^, the first and only FDA-approved interstitial therapeutic for GBM. We also provide considerations for the rational design of future implantable biodegradable materials along with the application of combination therapy to further improve current research endeavors.

## 2. Development and Overview of Gliadel

Currently, Gliadel^®^ is the only FDA-approved interstitial therapy available for GBM. Gliadel^®^ wafers are composed of the biodegradable copolymer poly[1,3-bis(p-carboxyphenoxy) propane-co-sebacic acid] p(CPP:SA) loaded with carmustine (BCNU), an alkylating agent derived from mustard gas [26,32]. In a Phase III clinical study, Gliadel^®^ increased median survival of patients to 58 weeks compared to 40 weeks for placebo [32]; however, side effects included worsening neurological deficits, seizures, CNS infection, and myelosuppression [33,34]. As the only FDA-approved biodegradable drug polymer composite for GBM, the research timeline and translational path informs similar research projects [35].

### 2.1. Gliadel^®^ Development

Gliadel^®^ development can be tracked based on papers and results published up to FDA approval. In the 1980s, the first step towards developing Gliadel^®^ was the application of the biodegradable polymer, p(CPP:SA), for the controlled release of a drug where the polymer and model drug, *p*-nitroaniline, were ground and compressed into a wafer [36,37]. In 1989, p(CPP:SA) (20:80) and (50:50) were found to be biocompatible in murine and rabbit brains, thus suggesting its applicability for drug delivery in this area [38,39]. Future evaluations of p(CPP:SA) were at a ratio of 20:80. This may have been due to the slower release kinetics of 50:50 being undesirable and the strong correlation between polymer degradation and drug release with 20:80 [36,37]. Pharmacokinetic characterization of p(CPP:SA) BCNU-loaded wafers was evaluated in rabbit brains and drug distribution was reported in 50%, 15%, and <10% of brain tissue after 3, 7, and 14 days post implantation, respectively, indicating that local delivery of BCNU in the brain provides high drug concentrations that are sustained over time [40]. Rats with intracranial tumors treated with BCNU-loaded p(CPP:SA) wafers had a significant increase in mean survival rate (62.3 days) compared to those treated with intraperitoneally administered BCNU (27.3 days) [41]. The BCNU-loaded p(CPP:SA) wafer was tested with and without radiation therapy in a non-human primate model, indicating biocompatibility of p(CPP:SA) polymer and verifying the safety of concomitant radiation therapy [42]. BCNU-loaded p(CPP:SA) wafers were compared with intratumoral injections of BCNU in a murine model with intracranial tumors; results found that treatment with wafers improved median survival time compared to 2 mg of BCNU injected intratumorally in rats (57.5 days compared to 21 days) [43]. In 1991, p(CPP:SA) and BCNU were spray dried into microparticles which were then compressed to form wafers and used in the first Phase I-II Clinical Trial of interstitial therapy. They reported that patients who received 3.85, 7.7, and 12.7 mg BCNU per wafer had a median survival times of 65, 47, and 23 weeks, respectively [26]. Analysis indicating why lower doses led to higher survival times was not made due to the small sample size, patients having different tumor types, and no cohort control. Results from a prospective, placebo-controlled study were published in 1995, showing that patients with GBM treated with BCNU-loaded p(CPP:SA) wafers had a 50% increase in 6-month survival compared to the placebo, and therefore it was concluded that interstitial chemotherapy provided a safe and effective method for treating recurrent malignant gliomas [44]. In 1996, BCNU-loaded p(CPP:SA) wafers were termed Gliadel^®^ and approved by the FDA for post-surgical GBM [43,45].

### 2.2. Current Clinical Use, Benefits, and Complications

Each Gliadel^®^ wafer is 1.4 cm in diameter, 1.0 mm thick, 200 mg in total mass with 7.7 mg of BCNU (3.85 wt%) [44,45]. The recommended application of Gliadel^®^ is to completely line the tumor resection cavity with up to a maximum of 8 wafers. It has been noted that Gliadel^®^ may result in seizures, intracranial hypertension, impaired neurosurgical wound healing, and meningitis.

A meta-analysis done by Chowdhary et al. analyzed survival and safety reports of 62 publications where patients with high-grade glioma were treated with Gliadel^®^ [46]. It was reported that Gliadel^®^-treated patients had a longer overall survival than those without the wafer and, specifically, those with newly diagnosed grade IV had a significant increase in median survival. The meta-analysis also indicated that patients treated with both Gliadel^®^ and TMZ had a higher overall survival compared to all other groups. This is due to BCNU and TMZ having similar mechanisms of resistance, where the combination can overwhelm high MGMT expression [47]. Similarly, a systematic literature review by Ashby et al. found that newly diagnosed high-grade glioma patients treated with Gliadel^®^ and the standard care (resection, radiation therapy, and oral TMZ) resulted in greater benefit than the standard of care or Gliadel^®^ alone [48].

While these studies demonstrate that Gliadel^®^ has improved therapeutic outcomes for GBM patients, the benefits are still only modest. This could be due to numerous factors, but a likely one is the utilization of BCNU as the anticancer agent. BCNU has low tumoricidal activity (requiring millimolar to micromolar concentrations for cytotoxic effect) [49], rapid clearance from the brain [40,50], and easily acquired drug resistance [51,52]. Additionally, although TMZ was not approved until 2005, it is much easier to administer (orally) and has the same mechanism of action as BCNU. As such, there is a large swath of GBM patients without an effective chemotherapy intervention because the cancer’s mechanism of action is not addressed with BNCU and TMZ. Paclitaxel (PTX), a more potent drug [53,54] with better pharmacokinetic properties [50] and a different mechanism of action than TMZ, was investigated during preclinical development [31], however, minimal drug was released from p(CPP:SA) wafers when scaled for non-human primates [55] because the surface area to volume changed with scaleup, modifying drug release kinetics adversely. This highlights the aspect of device design and the role that polymer and drug play in therapeutic efficacy.

A second factor in the modest benefit of Gliadel^®^ could be the relatively fast release of BCNU from the implant (lasting approximately 7 days). While BCNU dose was thoroughly investigated, the release rate of drug from the Gliadel^®^ implant was not optimized for maximum therapeutic benefit. Since then, our group has illustrated the importance of drug release rate on therapeutic outcomes. By changing the release rate, overall survival improved from 20% to 78% in a mouse model of GBM resection and recurrence [27].

Due to the high heterogeneity of GBM tumors, the ideal therapy will likely be a patient- and tumor-specific combination of drugs delivered at optimal release rates for each drug. Developing this future library of devices will require different polymers and formulations for each drug. The approach towards engineering such optimal interstitial therapy will be discussed in the upcoming sections of this review article.

## 3. Polymer

As the primary component of approaches like Gliadel^®^, polymer choice is a key consideration. Two of the most important features for brain drug delivery are polymer biocompatibility and biodegradability. Biocompatibility simply means that the polymer itself does not induce any adverse effects with brain tissue [56]. A polymer that may have the properties needed to release a drug locally when formulated must not cause any inherent side effects with the surrounding tissue. For a device to be inserted into the resection cavity, it is optimal to have used a polymer that is biodegradable so that surgery is not again needed to remove the carrier system. Therefore, the degradation by-products should be nontoxic metabolites that can be easily cleared [57]. If the polymer degrades at a constant rate, this can also facilitate and even dictate the rate of drug being released into the resection cavity and controlling this release is an important concept when trying to keep the drug concentrations at therapeutic levels while minimizing side effects.

**Figure 2:**
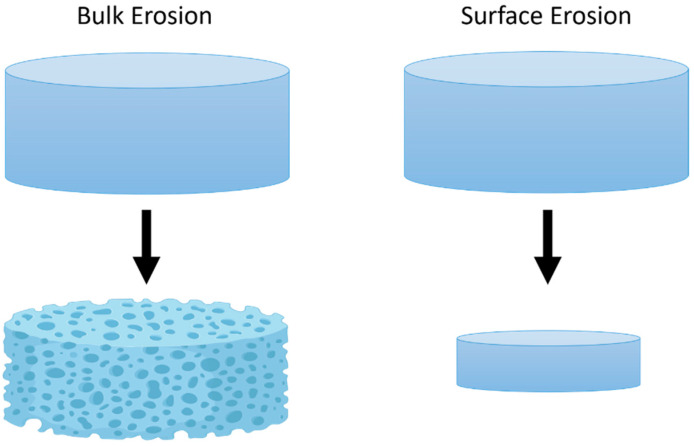
Simplified illustration of bulk erosion and surface erosion of compressed wafers.

The typical polymers used in the application of GBM therapy degrade via hydrolysis, which can occur by bulk or surface erosion (Figure 2) [58,59,60,61,62]. For a polymer to degrade, water must be in contact with the surface and, depending on the polymer’s hydrophilicity (or hydrophobicity), the water will hydrolyze the polymer at different rates. Surface erosion typically occurs with hydrophobic polymers since the water cannot permeate through the surface, and thus only hydrolyzes the surface and degrades the polymer at a linear rate. In the case of bulk erosion, polymer degradation does not follow a linear rate. This is primarily seen in copolymer systems that have monomers of varying hydrophilicity, are semicrystalline, and degrade into acidic by-products that autocatalyze the system.

### 3.1. Polyanhydrides

One of the first polymers formulated towards the development of biodegradable drug delivery devices were polyanhydrides, p(CPP:SA) [36,37,38,39]. p(CPP:SA) degrades by base-catalyzed hydrolysis where the degradation product is acetic acid, a natural metabolite present in humans and animals. It is a copolymer where the CPP component is hydrophobic and SA is hydrophilic, which allows the user to tune the degradation rate of the polymer by altering the ratio of the copolymer. Decreasing the CPP:SA ratio increases the rate at which the polymer degrades. During preclinical development for Gliadel^®^, p(CPP:SA) wafers were implanted in rat and rabbit brains, demonstrating biocompatibility [38,39,63]. To the best of author’s knowledge, p(CPP:SA) has been exclusively formulated via compression molding for drug delivery devices, as can be seen in Table 1.

### 3.2. Polyesters

Polyesters are one of the most widely used polymers in interstitial therapy for GBM. Polylactic acid (PLA), polyglycolic acid (PGA), and their copolymer form poly(lactic-*co*-glycolic) acid (PLGA), have been extensively studied as drug release systems. During degradation, PLGA is hydrolyzed at its ester linkages, leaving lactic acid and glycolic acid as its by-products. While these are both naturally occurring metabolites, they can lead to an increase in local acidity and autocatalyze the degradation of PLGA or alter drug activity [79,80,81]. The polymer’s degradation rates can be altered based on their stereochemistry and the ratio of lactic acid to glycolic acid in PLGA [82,83]. Lactic acid is more hydrophobic than glycolic acid, therefore, PLGA with a higher ratio of lactic to glycolic acid will degrade more slowly. When implanted into the brains of rats, polyester implants have shown low toxicity [84]. Polyester systems such as PLGA degrade by bulk erosion which can result in spontaneous dumping of drug and potential toxicity [85]. This was observed by Manome et al., where doxorubicin (DXR)-loaded PLGA sheets exhibited a sudden burst release of drug around 30 days [86]. The commercial availability of PLA and PLGA helps drive the extensive research towards its formulation for drug delivery.

Another polyester that has been used towards the research of interstitial therapy for GBM is polycaprolactone (PCL), which is also commercially available. The mode of degradation for PCL was found to be bulk erosion at physiological conditions (pH 7.4) but surface erosion at basic conditions (pH 13) [87,88]. The degradation rate of PCL is slow and can last up to several months. PCL has a semi-crystalline structure and when degrading, the crystallinity of a PCL device increases, which indicates that hydrolysis of the ester linkages occurs first at the amorphous regions [89]. The degradation kinetics of PCL are dependent on the molecular weight and crystallinity of the polymer; however, groups have developed copolymer systems with PCL to modify the degradation rate. Some of the copolymer systems used in devices for GBM include poly(ε-caprolactone-*co*-lactic acid) (PCL-LA) wafers [74], PCL-based polyurethane (PCL-Diol-b-PU) [90,91], and PCL crosslinked with poly(ethylene glycol) (PEG) (PCL-PEG-PCL) [92] electrospun nanofibers.

### 3.3. Acetalated Dextran

**Figure 3:**
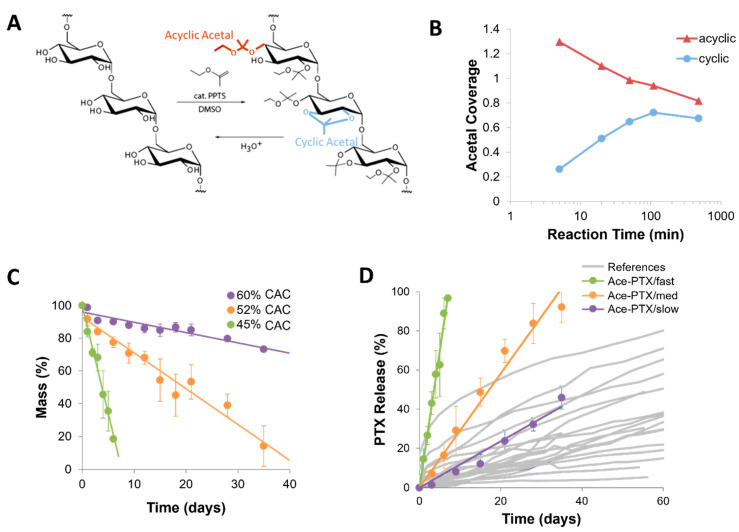
(**A**) Chemical synthesis reaction for converting dextran to Ace-DEX. (**B**) Reaction kinetics demonstrating the increase in cyclic acetal composition over time. Slightly modified and reproduced with permission from [94]. (**C**) Effect of cyclic acetal coverage (CAC) on degradation rate of Ace-DEX scaffolds. (**D**) Images of published in vitro release curves were analyzed using Automeris WebPlot Digitizer. Data were exported to excel and plotted together on the same x and y axis for ease of viewing. Grey lines are PTX release from polyester implants from references [28,29,30,77,78,95,96]. Red, green, and blue lines are PTX released from Ace-DEX scaffolds (Ace-PTX) with varying CAC. (**C**,**D**) Slightly modified and reproduced with permission from [27,94].

Acetalated dextran (Ace-DEX) is pH-sensitive biopolymer derived from the hydrophilic, polysaccharide dextran where the hydroxyl groups on the pendant are substituted with acetal groups [93,94]. By converting the hydroxyl groups into acetal groups, Ace-DEX becomes hydrophobic and degrades via acid-catalyzed hydrolysis as shown in Figure 3A. The degradation products of Ace-DEX are ethanol, acetone, and dextran (all pH neutral) which are all naturally occurring and/or metabolic by-products. Histological evaluation of murine brains implanted with Ace-DEX scaffolds showed no obvious toxicity [27,49]. Notably, Ace-DEX is acid sensitive, and has been shown to respond to the acidic microenvironment of GBM tumors with increased drug release [27].

With longer reaction times, the kinetically favored acyclic acetal converts towards a more thermodynamically stable cyclic acetal group (Figure 3B). The ratio of the cyclic to the acyclic acetal groups is termed the cyclic acetal coverage (CAC). Thus, the degradation rate of Ace-DEX can be tuned by controlling the degree of CAC. A higher CAC Ace-DEX has more cyclic acetals which are more stable to hydrolysis, thus higher CAC Ace-DEX degrades more slowly (Figure 3C). The range of Ace-DEX degradation is on the order of days to weeks, which is a faster range compared to polyesters (Figure 3D).

## 4. Formulation

Different methods have been used to encapsulate a drug into a device that has the capacity to be inserted into the tumor resection cavity. Just as drugs and polymers have physicochemical properties that can affect the release kinetics, different formulation methods have unique parameters that can change drug release rate. This section will introduce the three methods that have been commonly utilized to formulate drug delivery devices for GBM therapy: compression molding, electrospinning, and hydrogel synthesis.

### 4.1. Compression Molding

**Figure 4:**
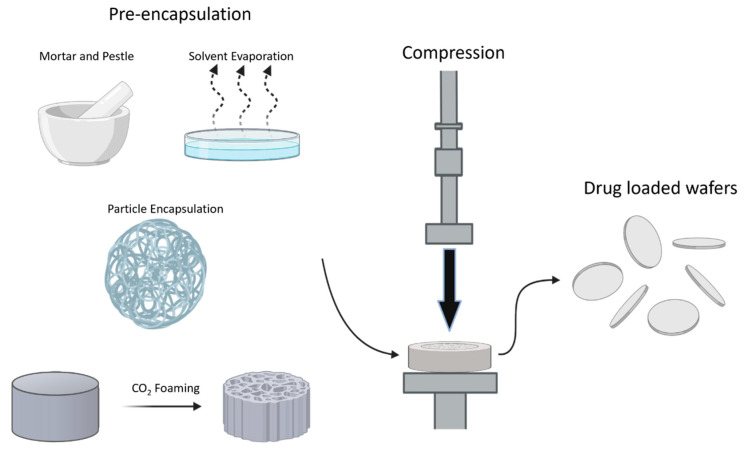
A schematic of fabricating drug-loaded wafers through compression molding where the pre-encapsulation methods used are using a mortar and pestle, solvent evaporation, encapsulating the drug in a particle, or creating a foam using supercritical CO_2_ foaming.

Compression molding is one of the first formulation methods used to encapsulate a drug in a polymer system for the interstitial treatment of GBM. Utilized in the development of Gliadel^®^ [44], compression molding is a simple method in which a drug and polymer are compressed together into a wafer using high pressure. Typically, prior to compression, the drug is first encapsulated within the polymeric matrix as a method of uniformly dispersing the drug. This can be achieved by spray drying, solvent casting, or blending via a vortex or mortar and pestle. The simplest pre-encapsulation method used is to blend the drug and polymer together. This can be done by directly mixing dry drug and polymer powders with a mortar and pestle (Figure 4). Alternatively, drug and polymer can also be dissolved in a solvent, creating a thin film after the solvent evaporates [31,64,65,66,67,68,69,70,72]. Supercritical CO_2_ is another way of mixing a polymer with the drug of interest to create a foam that can be compressed [30]. To first create the drug-loaded foam, a drug can be spray-dried in PLGA to develop particles which can be placed within a supercritical CO_2_ chamber under high pressure. This allows for the polymer and drug melt to dissolve into the CO_2_ gas and, as rapid decompression occurs, the CO_2_ escapes the polymer matrix leaving highly porous drug-loaded foams. Other methods of mixing or encapsulating a drug with a polymer have been listed in Table 1. For Gliadel^®^, the chemotherapeutic drug, BCNU, and the polymer, p(CPP:SA) (20:80), are formulated into particles using a spray drying method resulting in a powder texture which is then compressed into wafers [44].

### 4.2. Electrospinning

**Figure 5:**
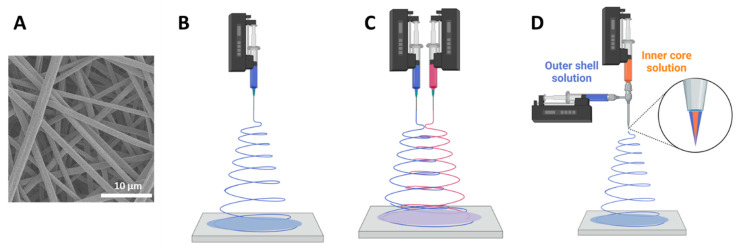
SEM image of an electrospun scaffold (**A**) and a simple schematic of the process of electrospinning (**B**) along with the nozzle for multi-axial (**C**) and co-axial electrospinning (**D**).

Electrospinning is another technique used to load a drug into a polymeric system that can result in a very thin mat composed of randomly or precisely aligned micro- or nano-fibers (Figure 5A) [97]. The process of electrospinning begins when a polymer and drug, dissolved in a solvent system, are slowly extruded from a syringe. A voltage differential is then applied between the needle of the syringe and a collector plate, breaking the surface tension of the polymer solution and forming a jet that accumulates as fibers on the collector plate (Figure 5B). Table 2 provides a list of devices formulated by electrospinning for GBM treatment. One advantage of electrospinning is that it can be a scalable process. More importantly, the high porosity created by the micro- or nano-fiber structure keeps the surface area to volume ratio high regardless of device size. This allows for an increase in the implant’s size with minimal effect on drug release kinetics. This is in contrast to compression molding wafers, where implant size can greatly affect device degradation and drug release. Additionally, by changing apparatus parameters (e.g., needle diameter, flowrate) and solution parameters (e.g., solvent system), the morphology of the composite’s fibers can be optimized to achieve a desired release rate. A discussion on how such parameters affect the release kinetics is provided in the Effect of Formulation Process on Release Kinetics section.

Modified methods of electrospinning such as co-axial or multi-axial electrospinning have been investigated to tailor different drug release results from these fibers [97]. Co-axial electrospinning is very similar to traditional electrospinning, the difference being the nozzle configuration (Figure 5D) where two solutions are pumped into the syringe but there is a compartment for the outer shell solution and another for the inner core solution. The two do not meet until they reach the tip of the needle. Multi-axial electrospinning is the simultaneous electrospinning of multiple syringes, each with a different drug–polymer system, towards one collector (Figure 5C). Using such a method could prove to be useful when trying to create a composite device that is loaded with multiple drugs, which may be necessary to better combat the high heterogeneity found in GBM tumors. This can also be an alternative to tailor different release rates for a single device [98]. Another strategy to create a composite device with multiple drugs is by creating multiple layers of separately electrospun composites adhered together with the use of a hydrogel [99].

**Table 2:** Drug delivery devices formulated via electrospinning. Details the polymeric material, drug encapsulated, needle setup, and final morphology.

Polymer	Drug	Needle	Morphology
Ace-DEX	DXR [49], Everolimus [54], PTX [27,54]	Uniaxial	Microfibers
PCL	Daunorubicin HCl [100], Methiopropamine [101], TMZ [98]	Uniaxial [100] Coaxial [101] Multiaxial [98]	Microfibers
PCL & alginate	TMZ & NGF [99]	Uniaxial	Multilayer fibers glued with gel
PCL & gelatin	Camptothecin [102]	Uniaxial	Nanofibers
PCL & PVP	MPA [101]	Coaxial	Microfibers
PCL-Diol-b-PU	TMZ [90,91]	Uniaxial	Microfibers
PCL-Diol-b-PU & chitosan	TMZ [90]	Uniaxial	TMZ loaded chitosan NP in fibers
PCL-PEG-PCL	Curcumin [92]	Uniaxial	Microfibers
PLA	DXR [49,103], PTX [27], TMZ [98]	Uniaxial [27,49,103] Multiaxial [98]	Microfibers
PLA-PEG	BCNU [104], DXR [103,105], PTX [105]	Uniaxial	Microfibers
PLA-PEO	Rapamycin [106]	Uniaxial	Nanofibers
PLGA	BCNU [107], Cisplatin [107], Combrestastatin [107], Irinotecan [107], PTX [28,29,95,108], TMZ [98]	Uniaxial	Nanofibers [98,107], Microfibers [28,29,95,108]
PPC & alginate	PTX [109], TMZ [109]	Uniaxial	Microparticles in microfibers
PVA	Dacarbazine [110]	Uniaxial	Nanofibers
PVP	Methiopropamine [101]	Uniaxial	Microfibers

Key terms: Ace-DEX: acetalated dextran; BCNU: carmustine; BuOH: butanol; DCM: dichloromethane; DMF: dimethylformamide; dichloromethane; DMSO: dimethylsulfoxide; DXR: doxorubicin; EtOH: ethanol; EVR: everolimus; HFIP: hexafluoro-2-propnaol; NGF: neuron growth factor; PCL: poly(ε-caprolactone); PDLLA: poly(D,L-lactic acid); PEG: polyethylene glycol; PEO: polyethylene oxide; PLA: poly(L-lactic acid); PLLA: poly(L-lactic acid); PTX: paclitaxel; TEA: triethylamine; TMZ: temozolomide;.

### 4.3. Hydrogel Synthesis

**Figure 6:**
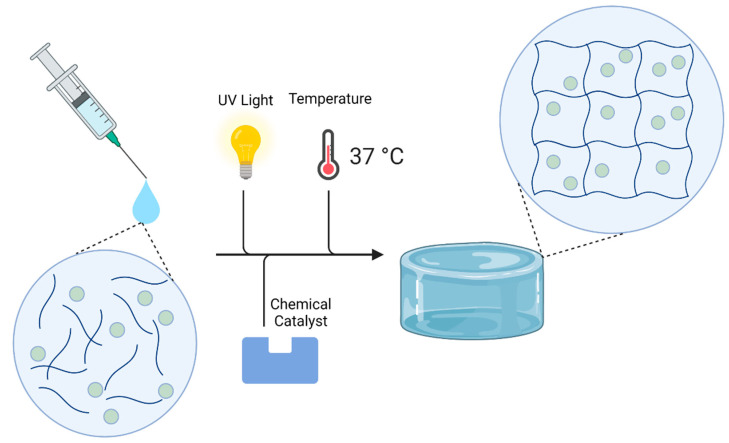
A syringe containing a hydrogel solution with a drug can be stimulated by UV light, temperature, or catalyst to begin the gelation process in situ.

Hydrogels are comprised of three-dimensional hydrophilic polymer networks and are another common delivery system utilized for GBM (Figure 6). Injectable biodegradable hydrogels provide a method of localized and sustained drug delivery at the tumor site. Their gelation process can occur either in situ or prior to injection. In situ gelation allows for the injectable solution to fill any target area shape, and more importantly, could allow for repeated dosing without the need for a second invasive procedure by simply injecting the hydrogel solution into the resection cavity. Alternatively, research has been done to refill the hydrogel depot with systemically administered prodrugs [111]. Hydrogel in situ crosslinking can be achieved by a chemical or physical stimulus [28,112,113,114,115]. Chemical or ionic stimuli can include the use of organic solvents or a catalyst, whereas physical crosslinking exploits temperature changes or light stimulation. Physical stimuli to crosslink hydrogels allow for liquid hydrogels to solidify while in contact with tissue and can allow for more contact surface area. Table 3 provides a list of hydrogels that have been used in the research for treatment options of GBM.

Ionic crosslinking allows for hydrogel formation prior to being implanted within the tumor site. One example of ionic crosslinking comes from Ranganath et al. where calcium was used to ionically link hydrogel beads composed of alginate [28,112]. Physical crosslinking is another method by which a hydrogel solution can begin the gelation process in situ. One physical crosslinking method that has been used for hydrogel formation is temperature. This hydrogel is a stable liquid below 37 °C, physiological temperature, but as it reaches normal body temperature, the solution begins to solidify into a gel. One example of this is OncoGel™, a thermosensitive triblock copolymer of PLGA-PEG loaded with PTX [124]. OncoGel™ reached Phase 2 clinical trials but was terminated due to business sponsor reasons rather than efficacy (ClinicalTrials.gov (accessed on 31 May 2021) identifier: NCT00479765). Another form of physical stimuli that can crosslink hydrogels is light. Photosensitive hydrogels can be injected into the tumor site in the form of a solution and once exposed to UV light, will form into a hydrogel [113,114,122]. Fourniols et al. studied poly(ethylene glycol) dimethacrylate that can entrap homogenized micelles of PEG-p(CL-co-TMC) loaded with TMZ [122]. By exposing the solution to UV light (λ = 400 nm) photopolymerization of the hydrogel can occur which resulted in ~45% burst release, followed by slow and sustained release of TMZ [122].

### 4.4. Combination Therapy

Treatment with a single drug is typically insufficient to combat cancer due to drug resistance and tumor heterogeneity. Combination therapy of two or more drugs with differing mechanisms of action can help delay the development of resistance and overcome existing drug resistance [129,130,131,132]. Identifying synergistic drug combinations, when two drugs have a greater combined effect than the sum of their separate effects, could further improve outcomes while minimizing drug dose and potential toxicity [131]. The recent Precision Medicine Initiative and The Cancer Genome Atlas (TCGA) program has led to a greater understanding of the genomic landscape of cancer and helped to identify new therapeutic targets [133]. In fact, GBM was fully sequenced in 2008 and investigators found that retinoblastoma protein (RB), tumor protein p53 (p53), and receptor tyrosine kinase/phosphatidylinositol 3-kinase (RTK/PI3K) pathways were deregulated in almost all GBM tumors [134]. Drugs that target these pathways offer a promising new therapeutic avenue for GBM.

**Figure 7:**
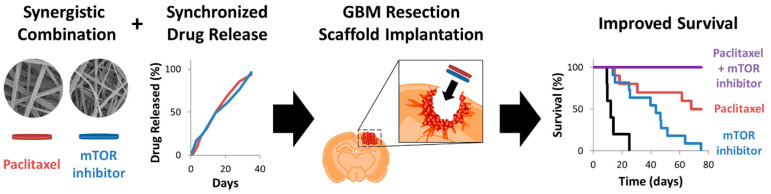
Summary of synergistic interstitial therapy in a resection and recurrence murine GBM model using PTX- and EVR-loaded Ace-DEX fabricated by electrospinning. Combination therapy resulted in 100% survival and prevention of tumor recurrence. Reproduced with permission from [54].

In current oncology practice, drug combination is most often achieved via systemic delivery of multiple chemotherapies or the addition of radiation or surgery. However, coordinating systemically administered drugs to maintain a synergistic ratio can be challenging due to different drug formulations, administration routes, and pharmacokinetic profiles, not to mention the potentially intolerable combined side effects. These obstacles could prevent optimal drug combination concentrations within the tumor. However, delivering drugs locally to a tumor offers more temporal control, and could allow for precise control of drug ratios. In fact, Recinos et al. found that delivering two drugs locally via P(CPP:SA) wafer resulted in improved outcomes than when one was administered orally. Specifically, they evaluated the effect of TMZ administration (either interstitially via P(CPP:SA) wafer or orally) in combination with BCNU-loaded P(CPP:SA) and radiation [64]. This study concluded that interstitial delivery of TMZ in combination with interstitial BCNU and radiation improved survival compared to orally administered TMZ combined with interstitial BCNU and radiation. This may be due to the coordinated dosing achieved through combined local delivery. This combination demonstrated improved efficacy despite BCNU and TMZ having the same mechanism of action. This has been shown in other studies, and authors hypothesize that it may be due to the depletion of MGMT, a DNA repair protein responsible for drug resistance. Similarly, utilizing the tunability of the biodegradable polymer acetalated dextran, our group formulated separate drug-loaded electrospun fibrous scaffolds with comparable release rates of two synergistic drugs (Figure 7) [54]. Therapeutic efficacy was tested in a murine model of GBM that included partial tumor resection. The combination of PTX and everolimus (EVR) scaffolds improved survival compared to controls and completely prevented post-surgical tumor recurrence. Table 4 below lists combination interstitial therapies delivered for GBM treatment detailing device design, in vitro release kinetics, and model for therapeutic efficacy.

While most studies were evaluated in orthotopic rodent models, some included surgical resection which best mimics clinical standard of care. This is an important consideration as surgical resection affects fluid flow in the brain and thereby drug diffusion from the device [135,136]. With the new therapeutic targets identified by TCGA, drug combinations offer a promising avenue to improve real world outcomes for GBM patients. Understanding the device design parameters discussed in the next section will help to achieve optimal drug release rates to facilitate clinical translation.

**Table 4:** Table of devices utilized in combination therapy for GBM treatment in murine models. Drugs used in combination as well as the design and formulation of each device are listed. When available, in vitro release rates of each drug are listed. Lastly, the animal models used for therapeutic efficacy are detailed, including tumor location, animal, whether tumor resection occurred, and the method of analysis (animal survival or tumor growth as monitored by bioluminescence).

Drugs	Device Design	In Vitro Release Kinetics	Model (outcome)	Ref
BCNU, TMZ	Co-loaded in compressed polymer wafer	BCNU: ----	Orthotopic, F344 rat no resection (survival)	[74]
		TMZ: linear release 100% at 35 days		
PTX, TMZ	PTX-loaded alginate microparticles electrospun into TMZ-loaded polymer fiber scaffold	PTX: linear release 100% at 7 days	----	[109]
		TMZ: linear release 100% at 5 days		
PTX, TMZ	PTX-loaded polymer microparticles incorporated in photopolymerizable hydrogel containing TMZ	----	Orthotopic, nude mouse, tumor resection (survival)	[114]
Plasmid DNA for RNAi of MMP2, PTX	Plasmid DNA-loaded polymer nanoparticles electrospun with PTX into polymer fiber scaffold	Plasmid DNA: ~15% release over 42 days	Orthotopic, nude mouse, no resection (tumor growth)	[108]
		PTX: ~10% release over 42 days		
EPR, PTX	PTX-loaded BSA nanoparticles incorporated in thermosensitive hydrogel containing EPR	EPR: ~80% at 12 days	Orthotopic, nude mouse, no resection (survival)	[118]
		PTX: ~50% at 12 days		
BCNU, CIS, CA-4 irinotecan	BCNU, CIS, and irinotecan electrospun into polymer fiber layer, followed by layer of CA-4 within polymer fibers	----	Orthotopic, Wistar rat, no resection (survival)	[107]
siRNA, MIT, CXCL10	siRNA loaded in MOF suspended in hydrogel containing MIT and CXCL10	siRNA: linear release 100% at 15 days	Orthotopic, C57BL6 mouse, no resection (survival)	[137]
		MIT: linear release 100% at 18 days		
		CXCL10: linear release 100% at 12 days		
BCNU, TMZ	Co-loaded in compressed polymer wafer	----	Orthotopic, F344 rat, no resection (survival)	[64]
PTX, EVR	Separately electrospun polymer fiber scaffolds	PTX: linear release 100% at 35 days	Orthotopic, nude mouse, tumor resection (survival)	[54]
		EVR: linear release 100% at 35 days		

Key terms: BCNU: carmustine; BSA: bovine serum albumin; CA-4: combretastatin; CIS: cisplatin; CXCL10: C-X-C Motif Chemokine Ligand 10; EPR: epirubicin; EVR: everolimus; MIT: mitoxantrone; MMP2: matrix metalloproteinase-2; PTX: paclitaxel; RNAi: interfering RNA; TMZ: temozolomide.

## 5. Controlling Release Kinetics

In order to apply synergistic drug combinations in interstitial therapy, understanding how drug properties, polymer properties, and formulation methods affect release kinetics is needed. Typically, to assess the release kinetics of the drug delivery system, the drug-loaded polymeric matrix is kept in phosphate buffer saline (PBS), at pH 7.4, 37 °C, and in constant shaking to mimic the human physiology. Ideally, drugs will be loaded in a biodegradable polymeric matrix where the burst release is minimized upon insertion of the device to minimize toxicity, the release of the drugs exhibit zero-order release kinetics, and the amount of drug released is controlled and sustained at predicted ratios to reach the synergistic effect. To meet this end, this section will discuss how the individual properties for drugs, polymers, and formulation methods affect the release kinetics of the polymeric drug delivery system.

## 6. Drug Properties on Release Kinetics

**Figure 8:**
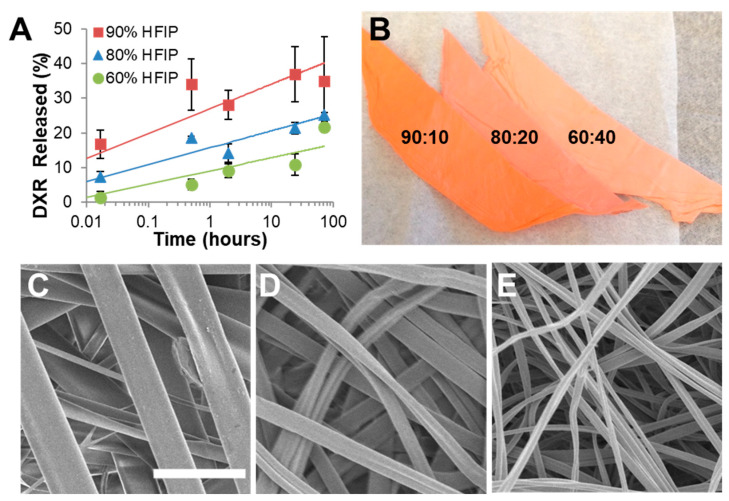
Effect of solvent system on drug solubility. (**A**) Burst release of DXR from Ace-DEX scaffolds electrospun with different HFIP to butanol ratios of 90:10 (black square), 80:20 (gray triangle), and 60:40 (white circle). (**B**) Picture of three different doxorubicin (DXR)-loaded Ace-DEX scaffolds electrospun with different solvent systems: hexafluoroisopropanol (HFIP) and butanol with ratios of 90:10, 80:20, and 60:40 (from left to right). Scanning electron micrographs of Ace-DEX/5Dox scaffolds electrospun in a solvent system of hexafluoroisopropanol (HFIP) and butanol with ratios of (**C**) 90:10, (**D**) 80:20, and (**E**) 60:40. Scale bar is the same for all images and represents 5 µm. Slightly modified and reproduced with permission from [49].

When designing the next generation of polymer and drug composites to treat GBM, careful considerations about a drug’s physicochemical properties should be kept in mind as these will affect the release rate from the device. Additionally, these same physicochemical properties will affect drug distribution in brain tissue after release. Drug release from a polymer matrix is controlled by diffusion and the degradation of the polymer. There are several physicochemical properties of a drug that may impact release kinetics, such as solubility. Solubility in this context refers to a drug’s maximum capacity to dissolve into the solution surrounding the device (typically an aqueous solution) at equilibrium and is dependent on its polymorphic form and polarity [138,139]. It is known that a concentration gradient typically drives diffusion, but it is also a function of solubility; a drug with higher solubility typically results in faster release and wider distribution if placed in an aqueous environment. Incomplete release of a drug can be exhibited if solubility is too low, but this can be compensated for by being incorporated into a polymer that erodes or degrades over time. By having the polymeric device degrade completely, the remaining drug is then released into the environment or medium. Solubility also plays a large role with the issue of supersaturation. This occurs when the drug loading exceeds the solubility for the system which can affect the release kinetics. Poor drug–polymer solubility can result in drug crystallization at the polymer surface and a burst release of the drug [140]. In contrast, good drug–polymer solubility allows the drug to be homogenously distributed throughout the polymer matrix and improves release kinetics [77]. This notion is further supported from the findings of Graham-Gurysh et al., where DXR was electrospun into a scaffold using three different solvent systems [49]. Burst release of DXR was decreased by decreasing the ratio of hexafluoroisopropanol (HFIP) to butanol (Figure 8A) [49]. It was also visually apparent that as the butanol content increases, DXR (a bright orange-colored drug) was more homogeneously incorporated into the polymer matrix rather than being stored near the surface of the fibers, as evidenced by a color change (Figure 8B) [49]. Solubility of a drug can also affect the rate of hydration and swelling of the polymer matrix, which in turn also affects drug release [141]. A highly soluble drug allows for a higher rate of water penetration within the device, resulting in a larger degree of swelling of the polymer matrix which then increases the rate of diffusion of the drug [141]. This is demonstrated by Hongtao et al., where a drug with the higher solubility resulted in a faster release from a hydrophilic polyethylene oxide (PEO) polymer matrix [141].

Another method of assessing solubility is with the partition coefficient (logP). LogP is the base-10 logarithmic value of drug partitioning between a hydrophobic solvent (octanol) to a hydrophilic solvent (water). This allows for a ranking of hydrophobicity or hydrophilicity between drugs. Knowing the logP of the drug, polymer, and solvent system used in the formulation process could provide insight on how compatible each component is with respect to the other. However, more importantly, knowing the logP of the drug can relate how the drug will partition into the interstitial fluid surrounding the device once it is implanted. A drug with a logP that indicates significantly high solubility in a hydrophobic solvent (>1) will release slowly from hydrophobic polymer, into an aqueous environment. In contrast, a drug with a logP that indicates it partitions into an aqueous environment (negative logP) will indicate that the drug will rapidly leave a hydrophobic polymer.

Zeng et al. compared the release of hydrophobic free-base DXR with hydrophilic DXR hydrochloride electrospun into a hydrophobic polymer (PLA) utilizing a chloroform and acetone solvent system [96]. Hydrophilic DXR hydrochloride formed crystals on fiber surfaces and had a burst release, whereas free-base DXR scaffolds displayed no surface crystals and a near zero-order release rate [96]. Fung et al., encapsulated three drugs with varying properties, BCNU (LogP = 1.5) [142], 4-HC (LogP = 0.3) [143], and PTX (LogP = 2.5) [144], within P(CPP:SA) wafers using compression molding [55]. PTX had markedly slower release rate compared to BCNU and 4-HC. The authors hypothesize that this was due to PTX having a much slower dissolution rate compared to its diffusion rate through the polymer. This notion is supported by the difference in logP values of PTX compared to BCNU and 4-HC.

Another important parameter is drug molecular weight (MW) and shape. In the classical Higuchi equation and Fickian diffusional release models, the rate at which a drug is released from a matrix is directly proportional to the diffusion coefficient [57,145]. In a system where it is assumed that diffusion is the primary source of drug release, it is seen that the diffusion coefficient is inversely proportional to the drug’s MW. This implies that by increasing the MW of a drug, the rate of diffusion decreases. The shape of the drug, which is the three-dimensional outline of space it fills, can also play a role on how it may interact with its environment [146]. Steric hindrance from the shape and size of the drug can affect the degree of hydrogen bonding and dipole–dipole interactions that can occur between the drug and polymer interface.

Polar surface area (PSA) can also affect diffusion as it can be considered a measurement of hydrogen bonding potential [147]. PSA is defined as the summed surface area of all polar atoms of a drug’s chemical structure (typically oxygen and nitrogen atoms and sometimes sulfur atoms) [148]. A study by Chaparro et al. found that rose bengal (PSA = 89.5 Å^2^) had a significantly slower release rate compared to rhodamine B (PSA = 52.8 Å^2^) from polycaprolactone (PCL) capsules [149]. It was hypothesized that the higher PSA of rose bengal compared to rhodamine B led to increased affinity with the ester groups of PCL, thus slowing the release rate of rose bengal [149]. While it is expected that rhodamine B, having the lower MW, to have a higher release rate, Chaporro et al. speculate that the higher PSA also contributed towards the substantial decrease in rose bengal’s release rate [149]. This is in agreement with the findings from Liu et al., where in modeling the release of six different drugs from hydroxyl pressure sensitive adhesives, a decrease in PSA correlated with an increase in drug release at lower drug loadings of around 0.25 wt% [147]. The number of hydrogen bond donors and acceptors can also affect the degree of hydrogen bonding that can occur between the polymer and drug. For example, a drug with more hydrogen bond donors can bond more readily to a polymer backbone that can readily accept hydrogen bonds, therefore reducing the release rate [149]. Therefore, a small molecule with a high PSA may release more slowly from a polymer implant than a similar molecule with a low PSA.

pKa is the negative base-10 logarithmic value of the acid dissociation constant (Ka) and also the pH that functional group(s) on the molecule are 50% ionized (unprotonated) and un-ionized (protonated) [148]. Typically, the un-ionized form of a drug is less hydrophilic, which may help it to diffuse more readily through membranes. Differences in drug pKa values can affect the degree of hydrogen bonding that can be achieved as a result of drug ionization. This in turn can affect the release kinetics of a drug from a polymeric system, where more hydrogen bonding between the drug and polymer interface will result in a slower release [147,149]. Furthermore, drug ionization has a significant impact on the drug’s solubility in the surrounding interstitial fluid and the body and therefore its release rate from the polymer system. The strength of the hydrogen bond has also been related to its pKa values [150]. Another intermolecular force to consider that could affect the release kinetics from a polymer matrix are dipole–dipole interactions between the drug and polymer interface. These interactions can be related to a drug’s polarizability which is a measurement of its ability to form instantaneous dipoles [151]. Liu et al. found that as polarizability increases, the drug release rate slows in hydroxyl pressure-sensitive adhesives [147]. This may be due to drugs with higher polarizability bonding more readily and strongly with the polymer matrix via dipole–dipole interactions. Therefore, when designing the optimal release rate for a drug delivery system, the pKa and intermolecular interactions the drug may have with the polymeric matrix should be considered.

Overall, the drug’s physicochemical properties all play a role in the release of the drug from the device due to both its solubility and interaction with the polymeric matrix. These are important design parameters to take into consideration when designing an interstitial GBM drug delivery system.

### 6.1. Polymer Properties on Release Kinetics

A polymer’s physicochemical properties will also affect the incorporation of a drug into its matrix and influence the drug’s diffusivity. Biodegradable hydrophobic polymers primarily release drug via diffusion and as the polymer degrades. By contrast, hydrophilic matrices, such as those applied in hydrogels, utilize the polymer swelling to allow for water to penetrate the hydrogel mesh and expand the pores to facilitate drug diffusion [152,153].

Many polymers have tunable degradation properties that allow for the user to change the rate of degradation and thus modify the release of the encapsulated drug. As noted in the previous section, Ace-DEX degradation can be tuned by CAC [27,54,93,94] ; p(CPP:SA) can be tuned through the ratio of p(CPP) to SA [36,37]; and PLGA can be changed through the ratio between lactic acid to glycolic acid [82,83]. The main takeaway is simply that controlling the ratio of a hydrophobic component to a hydrophilic component of the polymer can control the degradation rate overall.

Polymer properties that can affect diffusion are covalent and dipole interactions between the drug and/or the chains and physical entanglement between polymer chains. The molecular weight of a polymer can not only affect the formulation process when making a device but can affect the release kinetics of the drug. This was demonstrated by Omelczuk and McGinity who found that increasing molecular weight of PLGA tablets slowed the release rate of drug [154]. However, a change in molecular weight of the polymer may not always influence the release kinetics of the drug or the degradation rate of the device. Dang et al. varied the MW p(CPP:SA) polymer in Gliadel^®^ wafers and found it did not have an effect on its erosion kinetics or release of BCNU within the range of 48 to 110 kDa [155].

The polymeric backbone structure can also influence drug diffusion due to hydrophobic or hydrophilic interactions between the polymer matrix and drug. Lassalle and Ferreira were able to determine computationally that the hydrophobic and hydrophilic interactions between insulin and PLGA affected the rate of diffusion [156]. It was discerned that adding hydrophobic components to a hydrophilic backbone on the polymer allowed for a more pronounced hydrophobic interaction with non-polar residues of insulin. Overall, the polymer system utilized plays a pivotal role in device design, affecting implant degradation rate and compatibility with various formulation properties. Additionally, the chemical structure of the polymeric backbone will influence release kinetics through physicochemical interactions with the drug.

### 6.2. Effect of Formulation Process on Release Kinetics

The formulation process can affect how the drug is distributed within the polymeric matrix and the overall drug release kinetics, keeping in mind that high burst release can lead to unwanted local toxicity. Even within each fabrication system (i.e., compression molding, electrospinning, hydrogel) variables can be changed to further alter the rate at which the drug leaves the device.

In compression molding, the choice of pre-encapsulation method can affect drug release. Zembko et al. developed PLGA (50:50) wafers loaded with disulfiram (DSF) using compression molding and found that combining the drug and polymer by mortar and pestle at room temperature resulted in the formation of DSF crystals and a high burst release from the compressed wafer [76]. In contrast, by heat casting the mixture to 80 °C prior to compression molding, the formation of DSF crystals was prevented. This reduced the burst release of DSF and increased the release rate, as DSF was more evenly dispersed through the wafer and was more soluble than its crystalline form. Ong et al. compared the release profile foam formulated by supercritical CO_2_ to the compressed wafer form of it and found that the wafer resulted in a slower drug release rate than the foam [30]. This was likely due to the decrease in porosity when the foams were compressed into wafers, resulting in a lower surface area to volume ratio.

Electrospinning can be modified to alter drug release to minimize burst. Coaxial electrospinning can form a shell and core fiber which could allow for more precise control of drug release by adjusting the core:shell feed ratio. It has been shown that when drug is loaded within the core fiber, decreasing the feed flowrate of the core and keeping the feed flowrate of the shell constant results in the increase of shell thickness and a slower and more sustained release profile [101]. This method could be utilized to reduce burst release seen with many hydrophilic drugs to avoid unnecessary toxicity. In addition, coaxial electrospinning could be employed to achieve sequential drug release by loading one drug within the shell and another drug within the core. Multiaxial electrospinning was explored by Huang et al., who took a neuron growth factor (NGF)-loaded PCL membrane and adhered electrospun TMZ-loaded PCL fibrous membranes with an alginate hydrogel, creating a device which was cytotoxic to tumor cells while simultaneously facilitating nerve regeneration [99].

One challenge with electrospinning hydrophilic drugs within hydrophobic polymer systems is the compatibility between the drug, polymer, and solvent system. Hydrophilic drugs tend to result in a high initial burst release with less sustained drug release [90,103,105,109]. To mitigate this, some researchers have applied particle fabrication techniques to first encapsulate the drug(s) of choice before electrospinning [90,103,105,109]. Irani et al. used chitosan to encapsulate TMZ into nanoparticles via ionic gelation interaction prior to electrospinning within a synthesized poly (ε-caprolactone diol)-based polyurethane polymer [90]. Xu et al. used a water/oil emulsion technique to suspend PTX and DXR within poly(ethylene glycol)-poly(L-lactic acid) diblock copolymer [103,105]. This emulsion was then electrospun into a scaffold resulting in a slower release rate of the encapsulated drug compared to electrospinning without the emulsion.

For hydrogels, Ranganath et al. found that entrapping PTX in PLGA microspheres before the formation of alginate hydrogels decreased the initial burst release compared to alginate hydrogels without PLGA microspheres or PTX-loaded PLGA microspheres alone [112]. This is due to the dissociation of Ca^2+^ cross-links in the hydrogel matrix which then releases the microspheres. It was also shown that increasing microparticle packing resulted in slower PTX release and this can be attributed to the packing density of the particles that decreased the porosity of the hydrogel which hindered buffer penetration [112]. Arai et al. utilized another tactic, encasing DXR within a particle carrier system prior to encapsulation within thermosensitive hydrogel [127]. By incorporating the drug into a particle system, a sustained linear release is achieved whereas the drug being simply mixed into the hydrogel resulted in 100% of the drug released by day five [127]. This is due to hydrogel degradation releasing the particles first, with drug release from the particle through diffusion and degradation of the particle [127]. Additionally, Xu et al. showed that encapsulating a hydrophilic drug in an emulsion and electrospinning it into a hydrophobic polymer can decrease burst release and release rate [103]. Overall, this illustrates that multipart systems can help to mitigate poor release from hydrogels.

**Figure 9:**
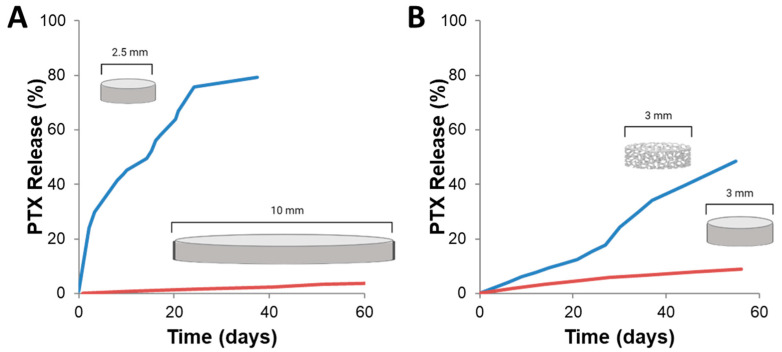
Effect of surface area to volume ratio on release of paclitaxel (PTX) from drug delivery devices. Images of published in vitro release curves were analyzed using Automeris WebPlot Digitizer. Data were exported to Excel and plotted together on the same x and y axis for ease of viewing. (**A**) PTX release from p(CPP:SA) (20:80) wafers. Blue lines from Walter et al. [31] is PTX release from 2.5 mm, 10 mg compressed wafers formulated for rats. Red line from Fung et al. [55] is PTX release from 10 mm, 200 mg compressed wafers formulated for primates. (**B**) PTX release PLGA wafers from Lee et al. [77] The blue line is ~3 mg porous foam wafer, 3 mm diameter × 1 mm tall. The red line is the same foam, compressed into a solid wafer ~8 mg with the same dimensions (3 mm diameter × 1 mm tall). (**A**) is slightly modified and reproduced with permission from [27].

Geometry of the drug delivery device can greatly influence drug release kinetics, specifically the change in surface area to volume ratio. This is most easily seen in scale-up for polymer wafers (Figure 9). PTX-loaded p(CPP:SA) wafer scaled for mice (10 mg, 2.5 mm) had markedly faster drug release compared to PTX-loaded p(CPP:SA) wafers scaled for non-human primates (200 mg, 10 mm). Additionally, by simply compressing a porous wafer, Ong et al. saw significant decrease in drug release rate from a PLGA wafer [30]. With electrospun scaffolds, fibers of varying sizes and geometry can be yielded to alter drug release. Fibers can take on a cylindrical or ribbon morphology which can alter a fiber’s surface area to volume ratio. By having smaller fibers, the surface area to volume ratio is increased, therefore it is expected that drug release will increase, as was seen by Chen et al. [157]. Fiber size and morphology can be altered by varying the concentration and viscosity of the polymer solution, solvent system, and flowrate of the ejected fiber [29,95,157,158]. However, some of these changes can also affect drug solubility within the system. For example, in Figure 8, changing the organic solvent system affected both fiber width and release rate of DXR. A higher percentage of HFIP generated wider fibers (Figure 8C–E), a higher burst release of DXR (Figure 8A), and brighter orange scaffolds (Figure 8B). So, although it would be expected that larger fibers would release a drug more slowly, the opposite is observed. In this case, the changes in drug and solvent system compatibility led to more DXR concentrated at the surface of the fiber rather than well distributed throughout, as evidenced through the brighter orange hue. This phenomenon overwhelmed any effect of the larger fiber diameter and resulted in faster DXR release rates.

Drug loading/content refers to the weight or mass percentage of the drug relative to the total mass of the device (wt%). Typically, a device with a higher drug loading will have a higher release rate and overall drug release based on when it reaches equilibrium [57,145]. This is due to the increase in concentration gradient between the drug in the device and the drug in the surrounding environment. Increasing the concentration gradient may also allow for further penetration into the brain, resulting in a larger distribution of drug. A study by Lesniak et al. showed that increasing the DXR drug loading in p(CPP:SA) (20:80) wafers resulted in a higher release rate and overall drug release into PBS [66]. An increase in drug content can also result in a higher burst release which can pose toxic dosage issues [140]. However, the sum effect of other drug properties can result in the opposite when increasing drug loading. For example, hydrophilic drugs such as DXR will lead to higher release rates but hydrophobic drugs may cause a decrease. In a study by Lee et al., increasing PTX drug loading in PLGA foams resulted in a decreased release rate due to PTX crystallizing in the PLGA polymer matrix from supersaturation [77]. Drug release, in this case, was dependent on the rate of dissolution of drug crystallites in PLGA rather than diffusion, polymer swelling, or polymer degradation [77]. If diffusion does not play a large role in drug release and instead the degradation of the polymer matrix is what facilitates release, then changing the loading may not affect the release rate [31,54]. It should also be noted that the method of analyzing the release of a drug from a device in %/day can provide different insight over mass/day. Yohay et al. showed that increasing riluzole loading in p(CPP:SA) wafers resulted in a lower percent release in 200 h but when converted to mass released, each wafer released the same amount of drug [68].

## 7. Conclusions

GBM is the most aggressive CNS tumor due to its high heterogeneity and invasiveness [4,5,6,7]. Despite advancements made in standard care, GBM still holds a poor prognosis but a large sum of preclinical research has been done to develop an innovative treatment solution [1,4]. One method of treatment that has been studied extensively for the past few decades is interstitial drug delivery via implantable biodegradable polymeric systems. While this strategy holds promise, further research is still required to increase therapeutic efficacy in order to translate to a clinical setting. Currently, the only form of interstitial therapy approved by the FDA for the treatment of GBM is Gliadel^®^. Gliadel^®^ is not universally accepted and is not part of the standard of care when it comes to GBM treatment, but it serves as an important benchmark for future interstitial therapeutic research to develop new interstitial therapies.

The design of GBM intestinal therapies should look towards optimizing drug release kinetics to reach proper drug combination ratios to combat the high heterogeneity and invasiveness of the cancer. Modelling drug distribution and tumor penetration can also hold high potential for therapeutic efficacy and give insight on whether drug concentration ratios within the brain are optimal for synergistic effects [159]. Since 90% of tumor recurrence is within 2 cm of the site of the original resected tumor [8], controlled and sustained release platforms delivered locally to the GBM resection site is needed. To do this, future research must consider how the complex interplay between the physicochemical properties of the drug and polymer of choice can affect the overall release kinetics as a result of intermolecular bonding as indicated from their pKa, PSA, and polarizability values. The method of formulation can also affect the release rate of the drug, which is affected by drug and polymer solubility and logP value.

## Figures and Tables

**Table 1:** Table of drug delivery devices formulated by compression molding for the treatment of GBM.

Polymer	Drug(s)	Pre-Encapsulation Method
p(CPP:SA)	4HC [55], BCNU [55,64], Camptothecin [65], DXR [66], Lactacystin [67], Memantine [68], PTX [31,55], Riluzole [68], Synthetic endostatin fragment [69], TMZ [64,70]	Solvent evaporation
p(CPP:SA)	Mitoxantrone [71]	Mix-melt
p(CPP:SA) and PLGA	BCNU [72], PTX [72]	Solvent evaporation
PLGA	ADS-I [73]	W/O/W double emulsion
	BCNU [74,75]	Vortex mix [75], solvent evaporation [74]
	DSF [76]	Mortar and pestle
	PTX [30,77]	Spray dried microparticles [77], Supercritical CO_2_ foaming [30]
	TMZ [74]	Solvent evaporation
PLGA and PEG	PTX [77]	Spray dried microparticles
p(DAPPG-EOP)	PTX [78]	In-line homogenizer to create microspheres
PCL-LA	TMZ [74]	Solvent evaporation

Key terms: ADS-I: ardipusilloside I; BCNU: carmustin; PCL-LA: poly(caprolactone-co-lactide); p(CPP:SA): poly[1,3-bis(p-carboxyphenoxy) propane-co-sebacic acid]; p(DAPG-EOP): polyphosphoester polymer; PLGA: poly(D,L-lactic-co-glycolic acid); PTX: paclitaxel; TMZ: temozolomide; 4-HC: 4-hydroperoxycyclophosphamide; W/O/W water in oil in water emulsion.

**Table 3:** Hydrogel drug delivery devices developed for the treatment of GBM. Details the hydrogel matrix, drug carrier system, drug encapsulated, and crosslinking method.

Hydrogel Matrix	Drug Carrier System	Drug	Crosslinking Method
Alginate	PLGA microparticles	PTX [28,112]	Ionic
Chitosan/glutaraldehyde	Alginate microparticles	TMZ [116], 131I [116]	Ionic
Chitosan/β-glycerophosphate	-	Ellagic acid [117]	Temperature
CMC-g-PNI PAAmMA/DTPAGd	BSA nanoparticles	EPI [118], PTX [118]	Temperature
Lipid nanocapsule	-	Gemcitabine [119,120,121], PTX [121]	Drug
P-CoFe_2_O_4_ NPs and PPZ	-	Irinotecan [115]	Temperature
PEG-DMA	PLGA nanoparticles	PTX [113,114], TMZ [114]	UV light
	PEG-p(CL-co-TMC) micelles	TMZ [122]	UV light
PLGA/ATEC/TEC	-	TMZ [123]	Plasticizer
PLGA/PEG	-	PTX [124]	Temperature
Thermoreversible gelation polymer	PLGA microparticles	CPT [125,126], DXR [127], VCR [125]	Temperature
	Liposome	DXR [127]	Temperature
	-	DXR [127,128]	Temperature

Key terms: ATEC: acetyl triethyl citrate; BSA: bovine serum albumin; CMC: carboxymethyl cellulose; CPT: camptothecin; DMA: dimethacrylate; DTPAGd: gadopentetic acid; DXR: doxorubicin; EPI: epirubicin; p(CL-co-TMC): poly(ε-caprolactone-co-trimethylene carbonate); PEG: polyethylene glycol; PLGA: polylactide-co-glycolide; PNIPAAmMA: poly(N-isopropylacrylamide-co-methacrylic acid); PPZ: poly(organophosphazene); PTX: paclitaxel; TEC: triethyl citrate; TMZ: temozolomide; VCR: vincristine.

## Data Availability

Not applicable.
